# Role of Circulating Microparticles in Impairing Endothelial Function and Activating Oxidative Stress Is Related to No‐Reflow in STEMI

**DOI:** 10.1155/cdr/9055462

**Published:** 2026-09-17

**Authors:** Wen-bo Li, Wei Zhang, Feng-Jun Chang, Zhe Li, Yu-Juan Yang, Gong Cheng, Xin Jiang

**Affiliations:** ^1^ Department of Cardiology, Shaanxi Provincial People′s Hospital, Xi′an, China; ^2^ Department of Cardiology, Honghui Hospital, Xi′an Jiaotong University, Xi′an, China, xjtu.edu.cn

**Keywords:** endothelial nitric oxide synthase, endothelial tube formation, no-reflow, ST-segment elevation myocardial infarction, vasodilatation

## Abstract

**Objective:**

Patients with ST‐segment elevation myocardial infarction (STEMI) frequently experience no‐reflow (NRF) during percutaneous coronary intervention (PCI). Nevertheless, the pathogenesis of NRF involves multiple factors and has not been fully elucidated to date. Given that endothelial dysfunction, inflammation, and oxidative stress are jointly implicated in both MP‐related processes and NRF occurrence, this study was performed to explore the potential role of circulating microparticles (MPs) in the development of NRF.

**Methods:**

MPs were isolated from healthy subjects (*n* = 30), patients with angina and negative coronary angiography (control, *n* = 30), and STEMI patients with (*n* = 30) or without NRF (*n* = 30). The effects of MPs on endothelium‐dependent vasodilatation, the expression of caveolin‐1 and endothelial nitric oxide synthase (eNOS), and the production of nitric oxide (NO), superoxide anion (O_2_^•−^), and intercellular adhesion molecule 1 (ICAM‐1) were detected in mice. Furthermore, we examined the effects of MPs on the proliferation and endothelial tube formation of human coronary artery endothelial cells (HCAECs), with or without *N* ^G^‐nitro‐L‐arginine methyl ester (L‐NAME) preincubation.

**Results:**

Compared with controls and healthy subjects, circulating MP levels were significantly higher in STEMI patients and were further elevated in STEMI patients with NRF. MPs isolated from NRF patients impaired endothelial‐dependent vasodilatation, decreased the expression of phosphorylated eNOS at Ser1177, reduced NO production, HCAECs proliferation, and endothelial tube formation, but upregulated the expression of caveolin‐1 and phosphorylated eNOS at Thr495, and increased the generation of O_2_^•−^ and ICAM‐1. The effects of MPs on HCAECs were partially blocked by L‐NAME.

**Conclusions:**

Circulating MPs are associated with NRF in the clinical context and induce endothelial dysfunction in experimental models. Our findings provide new insight into the underlying molecular mechanisms of NRF in STEMI in experimental models.

## 1. Introduction

ST‐segment elevation myocardial infarction (STEMI) is a severe clinical subtype of coronary artery disease (CAD), which can induce life‐threatening cardiovascular complications such as heart failure, cardiogenic shock, and even mortality [1]. The core clinical strategy for STEMI is to achieve rapid recanalization of infarct‐related arteries and restore blood perfusion to the infarcted myocardium. At present, percutaneous coronary intervention (PCI) is widely recognized as the most effective therapeutic modality for this condition [2]. No‐reflow (NRF) is defined as a significant reduction in coronary blood flow (TIMI flow Grade 0–1) that occurs in the absence of persistent occlusion, thrombosis, vasospasm, or severe residual stenosis at the initial target lesion following PCI [2]. When PCI is complicated by NRF, embolic particles induce mechanical obstruction through their mass effect, and simultaneously activate local coagulation pathways and inflammatory responses in the downstream microcirculation. In addition, the aggregation of leukocytes, platelets, and erythrocytes also contributes to the formation of intraluminal microvascular obstruction [3, 4].

Circulating microparticles (MPs) are small vesicles released by endothelial cells, platelets, leukocytes, and other vascular‐ or blood‐associated cells when the cells undergo activation or apoptosis [5, 6]. In prior research conducted by our group, hyperlipidemic model mice were administered with MP injection, and the results demonstrated that MPs exert adverse effects in multiple physiological and pathological processes of the organism, particularly in the progression of atherosclerosis and the promotion of inflammatory response [7]. In a subsequent study by our team, we injected MPs isolated from patients with acute coronary syndrome (with or without comorbid type 2 diabetes mellitus) into rats, and observed that MPs can induce the decoupling of endothelial nitric oxide synthase (eNOS) via inhibiting the eNOS–Hsp90 signaling pathway. This process upregulates the expression of caveolin‐1, which in turn reduces the production of nitric oxide (NO) and induces the generation of superoxide anion (O_2_^•−^), ultimately leading to the impairment of endothelial‐dependent vasodilation [8]. Nevertheless, existing evidence also indicates that MPs can exert protective effects under specific conditions, such as promoting neovascularization in ischemic tissues [9].

Given that MPs and nonobstructive CAD‐related myocardial ischemia (NRF) exhibit partial overlap in pathophysiological mechanisms, including endothelial dysfunction, inflammatory response, and oxidative stress; this study conducted a targeted investigation to clarify whether MPs are involved in the occurrence and progression of NRF by detecting indicators of endothelial function, oxidative stress, and inflammatory response.

## 2. Materials and Methods

### 2.1. Study Population

Between July 2023 and December 2023, the following participants were enrolled in this study: adult patients (including both male and female) with angina symptoms and negative coronary angiography results (*n* = 30), and STEMI patients undergoing PCI with or without nonobstructive CAD‐related myocardial ischemia (NRF), with 30 patients in each group. For STEMI patients diagnosed with NRF, the diagnostic and inclusion criteria were defined as follows: TIMI flow Grade 0–1 after opening the infarct‐related artery, with reflow restored to TIMI 3 grade and rapid normalization of elevated ST segment after administration of 50–300 *μ*g nitroprusside via microinfusion catheter, which ensured the drug reached the microvascular bed within 5 min after NRF onset; patients who received GP IIb/IIIa inhibitors or aspiration thrombectomy were excluded from this subgroup. Patients with conditions that may alter circulating MP levels were excluded from the study, including diabetes [10], infectious diseases [11], uncontrolled hypertension, renal insufficiency [12], severe trauma, heart failure [13], and patients who received surgical procedures within the past 3 months [14]. Potential confounding factors were identified and controlled through medical history collection, vital sign monitoring and laboratory examination, and patients that did not meet the eligibility criteria were excluded. A total of 30 healthy subjects matched for age and sex were enrolled as the blank control group. The clinical baseline characteristics of all participants are summarized in Table 1. All enrolled subjects provided signed written informed consent prior to study enrollment. This study protocol was reviewed and approved by the Ethics Committee of Shaanxi Provincial People′s Hospital. All study procedures were implemented in strict accordance with relevant guidelines and regulatory requirements: human‐related research procedures were performed in accordance with the Declaration of Helsinki, and in vivo animal experimental procedures followed the ARRIVE guidelines.

**Table 1:** Clinical characteristics.

	Healthy (*n* = 30)	Control (*n* = 30)	STEMI (*n* = 30)	STEMI + NRF (*n* = 30)
Age, yr	47.34 ± 9.56	46.53 ± 8.64	48.61 ± 9.25	47.81 ± 10.31
Male/female	16/14	15/15	15/15	14/16
Hypertension	0	8	10	11
Smoking	8	10	9	9
Family history	0	7	8	6
Culprit vessel				
LAD			10	11
LXC			5	3
RCA			15	16
TC, mmol/L	4.13 ± 1.14	4.32 ± 0.94	4.92 ± 1.61^∗^	5.01 ± 1.03^∗^
TG, mmol/L	1.21 ± 0.73	1.12 ± 0.68	1.62 ± 0.94^∗^	1.71 ± 0.84^∗^
HDL, mmol/L	1.02 ± 0.34	1.01 ± 0.31	0.96 ± 0.27	0.98 ± 0.36
LDL, mmol/L	3.06 ± 0.86	2.92 ± 1.13	3.45 ± 0.98^∗^	3.39 ± 1.25^∗^
Medications				
*β*‐receptor blockers	0	25 ^∗^	24 ^∗^	26 ^∗^
Statins	0	30 ^∗^	30 ^∗^	30 ^∗^
Antiplatelet	0	30 ^∗^	30 ^∗^	30 ^∗^

*Note:* Values are listed as means ± SD.

Abbreviations: HDL, high density lipoprotein; LAD, left anterior descending artery; LDL, low‐density lipoprotein; LXC, left circumflex artery; RCA, right coronary artery; TC, total cholesterol; TG, triglyceride.

^∗^versus control or healthy, *p* < 0.05.

### 2.2. MPs Extraction

Venous blood samples were collected from four groups of participants: blank group participants, angina control group participants, STEMI patients without NRF phenomenon during PCI, and STEMI patients with NRF during PCI (for the latter group, sampling was performed immediately after coronary blood flow restoration). All samples were collected through an indwelling needle inserted into the left arm. MPs were isolated by centrifugation (Beckman, California, United States) in accordance with the protocol reported in previous literature [8]. The specific procedure was as follows: Firstly, after the initial centrifugation of blood samples at 11,000 × g and 4°C for 2 min, the supernatant was collected to obtain platelet‐poor plasma. Secondly, the platelet‐poor plasma (was further centrifuged at 13,000 × g and 4°C for 45 min, and the sediment was collected to obtain MP pellets. Finally, the isolated MPs were resuspended in 100 *μ*L of RPMI 1640 medium (Gibco‐Invitrogen, Carlsbad, California), stored at −80°C, and used within 3 weeks. MP concentrations were determined (in technical triplicate using the bicinchoninic acid protein assay (Merck). Considering the limited sample size in this study, triplicate experiments were conducted to guarantee the reproducibility of experimental results and control for technical errors. Given that only a limited volume of venous blood (3–5 mL per participant) could be collected from each participant, which was insufficient to provide the MP quantity required for subsequent experiments, MPs from five participants in the same group were pooled to meet the sample requirements for subsequent assays, resulting for 30 participants were enrolled, in approximately 6 biological replicates for each subsequent experiment.

### 2.3. Vasodilatation Detection

Specific pathogen‐free C57BL6 mice (8 weeks of age, body weight:25 ± 2 g) were anesthetized via intraperitoneal injection of pentobarbital at a dose of 10 mg/kg (purchased from Sigma–Aldrich) [15]. After euthanasia, the thoracic aorta was rapidly isolated, and surrounding connective and adipose tissues were carefully removed in prewarmed Krebs solution continuously aerated with a gas mixture of 95% O_2_ and 5% CO_2_. The isolated thoracic aorta was subsequently cut into vascular rings of 3–5 mm in length. Each vascular ring was mounted on an isometric force transducer (AD Instruments Co., Australia), then placed in organ baths filled with aerated Krebs solution (95% O_2_ and 5% CO_2_) and maintained at 37°C for 30 min to reach a baseline equilibrium. After baseline stabilization, repeated contractile stimuli with 60 mmol/L KCl (purchased from Sigma–Aldrich) were applied at least three times to stabilize vascular tension. Subsequently, the aortic rings were incubated for 30 min with 3 mg MPs isolated from the corresponding experimental group. After incubation, the aortic rings were preconstricted with 10^−6^ mol/L phenylephrine (PE, purchased from Sigma–Aldrich). At 2 min after the preconstriction reached a stable plateau, cumulative dose‐dependent vasorelaxation responses were induced by adding acetylcholine (Ach) at concentrations ranging from 10^−8^ mol/L to 10^−4^ mol/L (purchased from Sigma–Aldrich), and relaxation responses were recorded. To verify that the observed relaxation response was endothelium‐dependent, additional aortic rings were preincubated with 1 mmol/L *N* ^G^‐nitro‐L‐arginine methyl ester (L‐NAME, purchased from Sigma–Aldrich) for 30 min before the vasorelaxation assay was performed. L‐NAME was added to all groups (including healthy and control) at the same concentration and incubation time. Data are from mouse aortic rings treated ex vivo with patient‐derived MPs, not from in vivo animal models of STEMI or NRF.

### 2.4. Western Blot Analysis

Following 1 h of incubation with RPMI 1640 medium or 3 mg of MPs isolated from each experimental group, total proteins were extracted from the thoracic aorta. Western blot analysis was subsequently performed to detect the protein expression of caveolin‐1 (Cat No. 3267, Cell Signaling Technology, Beverly, Massachusetts, United States), eNOS (Cat No. sc‐654, Santa Cruz Biotechnology, Santa Cruz, California, United States), as well as eNOS phosphorylation at sites Ser1177 (Cat No. 9571, Cell Signaling Technology, Beverly, Massachusetts, United States), and Thr495 (Cat No. 9574, Cell Signaling Technology, Beverly, Massachusetts, United States).

### 2.5. Superoxide (O_2_^•−^) Detection

Following 1 h incubation with RPMI 1640 medium or 3 mg of MPs from each experimental group, the thoracic aorta was isolated and adherent adipose tissue was trimmed away. The aorta was then split longitudinally and incubated with hydroethidine (HE, 10 *μ*M, Sigma–Aldrich) for 30 min. Fluorescence images were subsequently captured using a fluorescence microscope, and quantitative image analysis was performed using NIH ImageJ software. Hydrogen peroxide (H_2_O_2_, 0.5 M, Sigma–Aldrich) was applied as a positive control (merely assay validation controls), with three technical replicates set for each experimental group.

### 2.6. NO Detection

After 1 h incubation with RPMI 1640 medium or 3 mg of MPs from each experimental group, the thoracic aorta was isolated, and the surrounding adipose tissue was surgically removed. Following longitudinal dissection, the aorta was incubated with diaminofluorescein diacetate (DAF‐2DA, 10 *μ*M, Calbiochem). Fluorescence images were acquired via fluorescence microscopy, and quantitative image analysis was conducted using NIH ImageJ software. Vascular endothelial growth factor (VEGF, 50 ng/mL, Sigma–Aldrich) was utilized as the positive control (merely assay validation controls), and three technical replicates were set for each experimental group.

### 2.7. Intercellular Adhesion Molecule 1 (ICAM‐1) ELISA Detection

After 1‐h incubation with RPMI 1640 medium or 3 mg of MPs isolated from each experimental group, the cell culture supernatant was collected for the quantification of ICAM‐1 via enzyme‐linked immunosorbent assay. Briefly, 100 *μ*L of the obtained supernatant was coincubated with an ICAM‐1 monoclonal antibody (Sigma–Aldrich) in a 96‐well microplate for 30 min. Following this incubation period, horseradish peroxidase (HRP)‐conjugated secondary antibodies were added to the plate and incubated for an additional 30 min. Finally, after the addition of 3,3 ^′^,5,5 ^′^‐tetramethylbenzidine (TMB) HRP substrate, the concentration of ICAM‐1 was measured at an absorbance wavelength of 450 nm using a microplate reader (Safire, Tecan Infinite 200). Three technical replicates were set for each experimental group.

### 2.8. Proliferation Detection

Primary human coronary artery endothelial cells (HCAECs, ScienCell Research Laboratories, Cat. No. #6020) were seeded into 96‐well culture plates and cultured until the cell density reached approximately 50% confluence. Endothelial cell medium (ECM, ScienCell, Cat. No. #1001) supplemented with 1% fetal bovine serum, containing either 1 mM L‐NAME (Sigma–Aldrich) or no L‐NAME, was added to the wells, and cells were preincubated for 30 min. Subsequently, RPMI 1640 medium or 3 mg MPs was added, followed by 24 h of incubation. After three washes with phosphate‐buffered saline (PBS), cells were incubated with Cell Counting Kit‐8 (CCK‐8, a commercial cell proliferation detection kit) for 2 h. The absorbance at 450 nm (OD value) was then measured with a microplate reader to evaluate cell proliferation, with three technical replicates set for each experimental group.

### 2.9. Endothelial Tube Formation Detection

The preparation procedures were conducted as follows: First, Matrigel was mixed with an equal volume of precooled Dulbecco′s Modified Eagle Medium (DMEM), after which the mixture was added to a precooled 48‐well cell culture plate. After gentle homogenization via shaking, the 48‐well plate was transferred to a cell culture incubator and incubated for 20 min to allow the Matrigel to solidify completely. Next, HCAECs at a concentration of 10^5^ cells/mL, with a volume of 300 *μ*L per well, were inoculated into the solidified gel. The cell suspensions were prepared both with and without the addition of 1 mM L‐NAME (purchased from Sigma–Aldrich), followed by a 30‐min incubation. Finally, 3 mg of MPs or an equal volume of RPMI 1640 medium was added to each well, and the system was incubated for an additional 12 h. After incubation, images of formed endothelial tubes were captured for subsequent quantitative analysis. Three technical replicates were set for each experimental group.

### 2.10. Flow Cytometry Analysis

Flow cytometry was employed to identify the cellular origin of MPs following the protocol reported in previous literature [16]: 20 *μ*L of FITC‐labeled anti‐CD41 antibody and PE‐labeled anti‐CD31 antibody or their corresponding isotype control (Beckman Coulter) were mixed with 50 *μ*L of ethylenediamine tetraacetic acid (EDTA)‐anticoagulated platelet‐poor plasma in a 12 × 75 mm test tube. After thorough mixing, the reaction system was incubated at room temperature in darkness for 30 min. Subsequently, 20 *μ*L of flow cytometry calibration beads (Beckman Coulter) with known concentrations provided by manufacturer were added into the antibody‐labeled sample tube for subsequent counting and analysis, with the gate threshold set to < 1 *μ*m. In this study, endothelial‐derived MPs (EMPs) were defined as the CD31(+)/CD41(‐) MP population, whereas CD31(+)/CD41(+) MPs were identified as platelet‐derived MPs (PMPs).

### 2.11. Statistical Analysis

Data analysis was performed using SPSS 20.0 statistical software, and visualization plotting was completed with GraphPad Prism 5.0 software. The normality of the distribution of all variables was tested via the one‐sample Kolmogorov–Smirnov test. Quantitative data were expressed as mean ± standard deviation (mean ± SD). For comparisons among multiple groups, one‐way analysis of variance was adopted, and Bonferroni multiple comparison test was further performed for pairwise comparisons between groups. Differences were defined as statistically significant when *p* < 0.05.

## 3. Results

### 3.1. Clinical Data

Except for total cholesterol (TC), triglyceride (TG), low‐density lipoprotein (LDL) levels (the correlation analysis between lipid levels and MPs content did not find any correlation between them, data not shown), and medication, all clinical characteristics were similar among all groups (Table 1).

### 3.2. MPs Concentration

**Figure 1:**
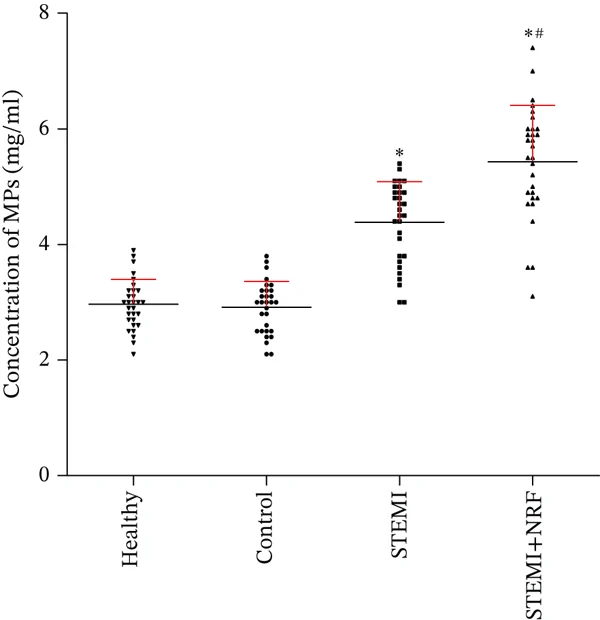
Concentration of MPs. Compared with those in the healthy (3.04 ± 0.21 mg/mL) and control (3.08 ± 0.63 mg/mL), the MP concentrations were greater in STEMI patients (4.38 ± 0.83 mg/mL). In addition, MP concentrations were further increased in STEMI patients with NRF (5.41 ± 0.17 mg/mL). The data are presented as the means ± SD,  ^∗^ versus control and healthy; # versus STEMI, *p* < 0.05.

Compared with the healthy (3.00 ± 0.40 mg/mL, *n* = 30, Figure 1) and control (3.02 ± 0.38 mg/mL, *n* = 30, Figure 1), MP concentrations were increased in STEMI patients (4.38 ± 0.70 mg/mL, *n* = 30, Figure 1). MPs concentrations were significantly increased in STEMI patients with NRF (5.43 ± 0.97 mg/mL, *n* = 30, Figure 1). Thus, MPs are stimulated by STEMI and further enhanced when NRF was present.

### 3.3. Effects of MPs on Endothelium‐Dependent Vasodilatation

**Figure 2:**
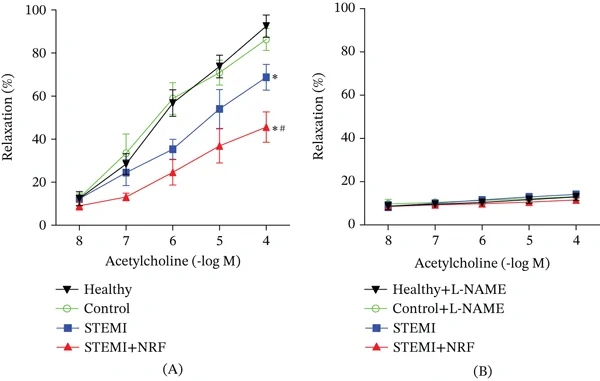
Effects of MPs on endothelium‐dependent vasodilatation. (A) Compared with those in the healthy and control groups, MPs from STEMI patients impaired endothelium‐dependent relaxation and were further impaired by MPs from NRF during PCI. (B) All aortic rings were preincubated with 1 mmol/L L‐NAME for 30 min prior to the relaxation assay. The data are presented as the means ± SD;  ^∗^ versus control and healthy, # versus STEMI; *p* < 0.05. (Data are from mouse aortic rings treated ex vivo with patient‐derived MPs, not from in vivo animal models of STEMI or NRF).

To detect the effect of MPs on vasodilatation, aortas from C57BL6 mice were isolated and treated with MPs. As shown in Figure 2, compared with those in both the healthy and control groups, MPs from STEMI patients impaired endothelium‐dependent relaxation, and this effect was enhanced by MPs from STEMI patients with NRF during PCI (Figure 2A). All of these effects of MPs were largely blocked by preincubation with L‐NAME (Figure 2B), which indicated that MPs mainly impaired endothelium‐dependent relaxation.

### 3.4. Effects of MPs on the Expression of Caveolin‐1 and eNOS Phosphorylation Sites

**Figure 3:**
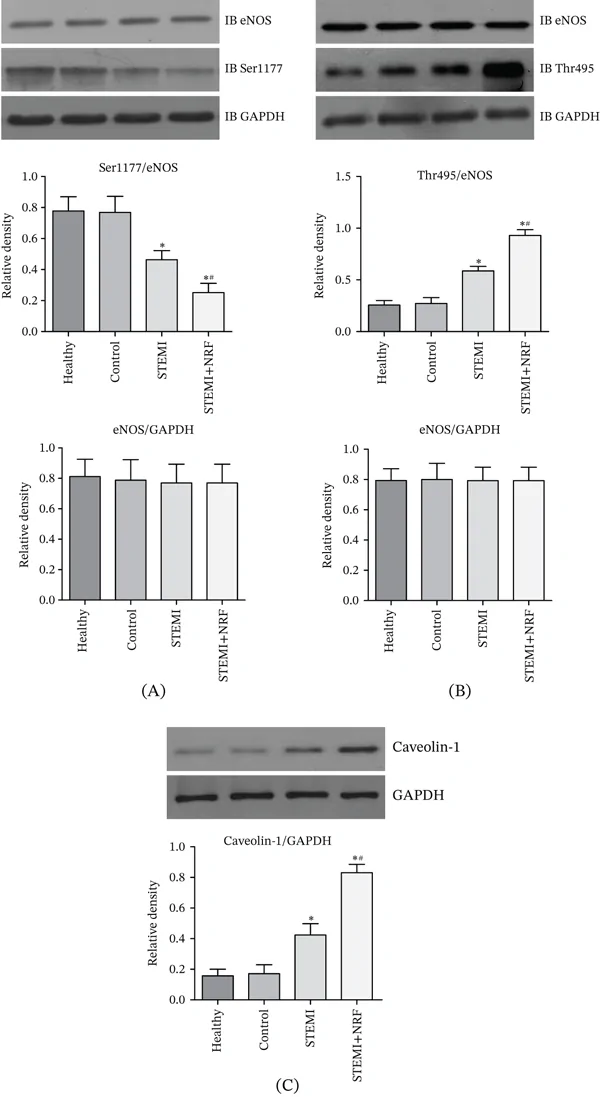
Effects of MPs on the expression of the caveolin‐1 and eNOS phosphorylation site. (A) Compared with healthy and control groups, MPs from STEMI patients (especially those with NRF) reduced the expression of the eNOS phosphorylation site at Ser1177 (which promotes the activation of the eNOS protein). (B) MPs from STEMI patients (especially those with NRF) increased expression of the eNOS phosphorylation site at Thr495 (which promotes the inactivation of the eNOS protein). (C) MPs from STEMI patients (especially those with NRF) increased expression of caveolin‐1. The data are presented as the means ± SD;  ^∗^ versus control and healthy, # versus STEMI; *p* < 0.05.

To investigate the mechanism by which MPs affect vascular function, the expression of caveolin‐1 and eNOS were detected with western blot. Compared with those from healthy and controls, MPs from STEMI patients reduced the expression of the eNOS phosphorylation site at Ser1177 (Figure 3A) but increased the expression of caveolin‐1 and eNOS phosphorylation site at Thr495 (Figure 3B,C). Moreover, these effects were enhanced by MPs from STEMI patients with NRF during PCI (Figure 3). Therefore, MPs affect vascular function by inactivating the eNOS signaling pathway.

### 3.5. Effects of MPs on NO and O_2_^•−^ Generation

**Figure 4:**
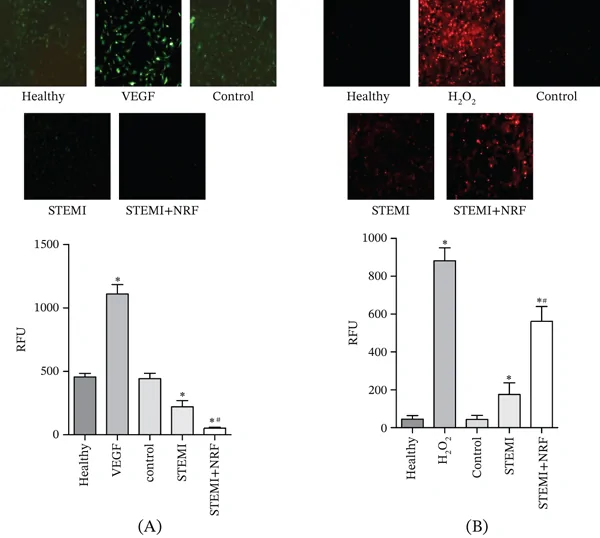
Effects of MPs on NO and O_2_^•−^ generation. (A) Compared with those from the healthy and control samples, MPs from the STEMI samples (especially those with NRF) decreased NO. (B) MPs from the STEMI samples (especially those with NRF) increased O_2_^•−^ generation. The data are presented as the means ± SD;  ^∗^ versus control and healthy, # versus STEMI; *p* < 0.05.

To investigate whether MPs impair endothelial function by increasing oxidative stress, the effects of MPs on NO and O_2_^•−^ generation on aortas were detected. Compared with the MPs from the healthy and control samples, the MPs from the STEMI patients presented with decreased NO levels (Figure 4A) but increased O_2_^•−^ generation (Figure 4B). Moreover, MPs from STEMI patients with NRF during PCI further decreased NO and increased O_2_^•−^ generation (Figure 4). Therefore, MPs may impair endothelial function by increasing oxidative stress.

### 3.6. Effects of MPs on ICAM‐1 Levels

**Figure 5:**
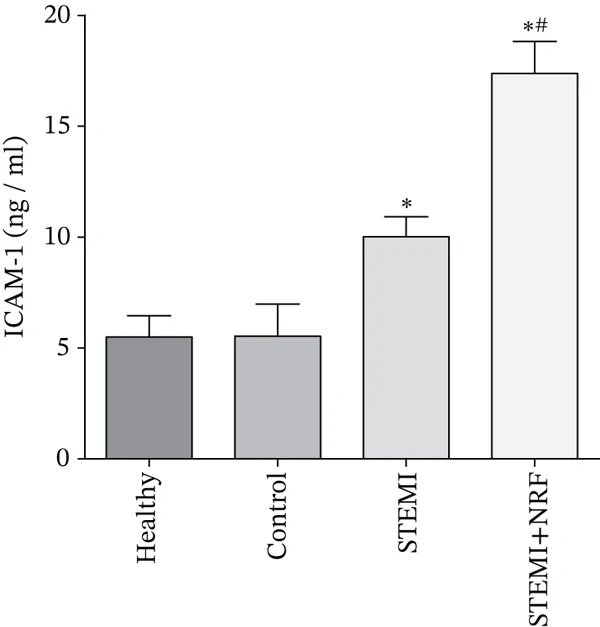
Effects of MPs on ICAM‐1 levels. Compared with the healthy controls and controls, MPs from STEMI patients (especially those with NRF) had increased ICAM‐1 levels. The data are presented as the means ± SD;  ^∗^ versus control and healthy, # versus STEMI; *p* < 0.05.

The levels of ICAM‐1, a cytokine of inflammation and downstream of oxidative stress, in mice aortas were detected via ELISA to detect whether MPs affect endothelial function through inflammatory response. Compared with the MPs from the healthy and control samples, the MPs from the STEMI patients had increased ICAM‐1 levels (Figure 5). Moreover, these effects were enhanced by MPs from STEMI patients with NRF during PCI (Figure 5). The influence of MPs on ICAM‐1 further confirmed that MPs may impair endothelial function by increasing oxidative stress.

### 3.7. Effect of MPs on Proliferation

**Figure 6:**
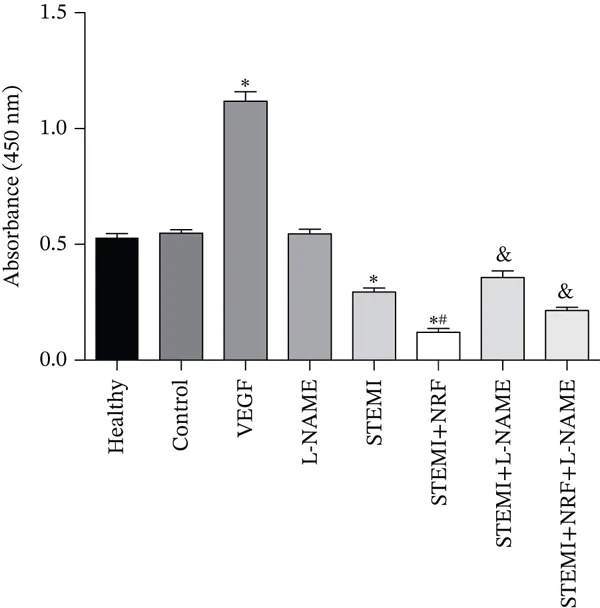
Effects of MPs on proliferation. VEGF can stimulate HCAECs proliferation, and MPs from STEMI patients (especially those with NRF) can inhibit HCAECs proliferation. Preincubation with L‐NAME (an inhibitor of eNOS) partly blocked the effect of MPs on HCAECs proliferation. Data are presented as the means ± SD;  ^∗^ versus control and healthy, # versus STEMI, & versus STEMI and STEMI + NRF; *p* < 0.05.

To evaluate the impact of MPs on the long‐term prognosis (such as angiogenesis in the infarcted area) of patients, we examined the effect of MPs on endothelial cell proliferation and tube formation. Compared with the healthy and control, MPs from STEMI patients (especially those with NRF during PCI) inhibited the proliferation of HCAECs (Figure 6). However, the ability of MPs to affect HCAECs proliferation was partly blocked by L‐NAME (an inhibitor of eNOS; Figure 6). As the initial step of angiogenesis (which may affect the repair of noninfarcted myocardium and the prognosis of STEMI patients), the proliferation of HCAECs is partly inhibited by MPs from STEMI patients, with or without NRF.

### 3.8. Effect of MPs on Endothelial Tube Formation

**Figure 7:**
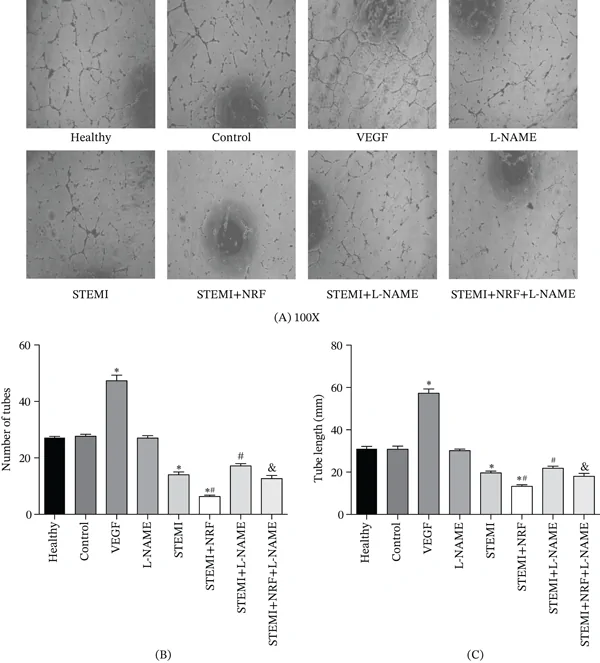
Effects of MPs on endothelial tube formation. (A) Original image of endothelial tube formation; (B) numbers of vessels; (C) vessel length. VEGF can stimulate HCAECs tube formation, and MPs from STEMI patients (especially those with NRF) can inhibit HCAECs proliferation. Preincubation with L‐NAME (an inhibitor of eNOS) partly blocked the effect of MPs on HCAECs proliferation. Data are presented as the means ± SD;  ^∗^ versus control and healthy, # versus STEMI, & versus STEMI and STEMI + NRF; *p* < 0.05.

Compared with that in the healthy and control groups, the ability of MPs from STEMI patients (especially those with NRF during PCI) to stimulate HCAECs tube formation was weakened. The inhibiting effects of MPs on HCAECs tube formation could be partly blocked by an inhibitor of eNOS (L‐NAME, Figure 7). The inhibitory effects of MPs from STEMI patients with or without NRF on endothelial tube formation may lead to cardiac dysfunction and poor prognosis.

### 3.9. Origin of the MPs

**Figure 8:**
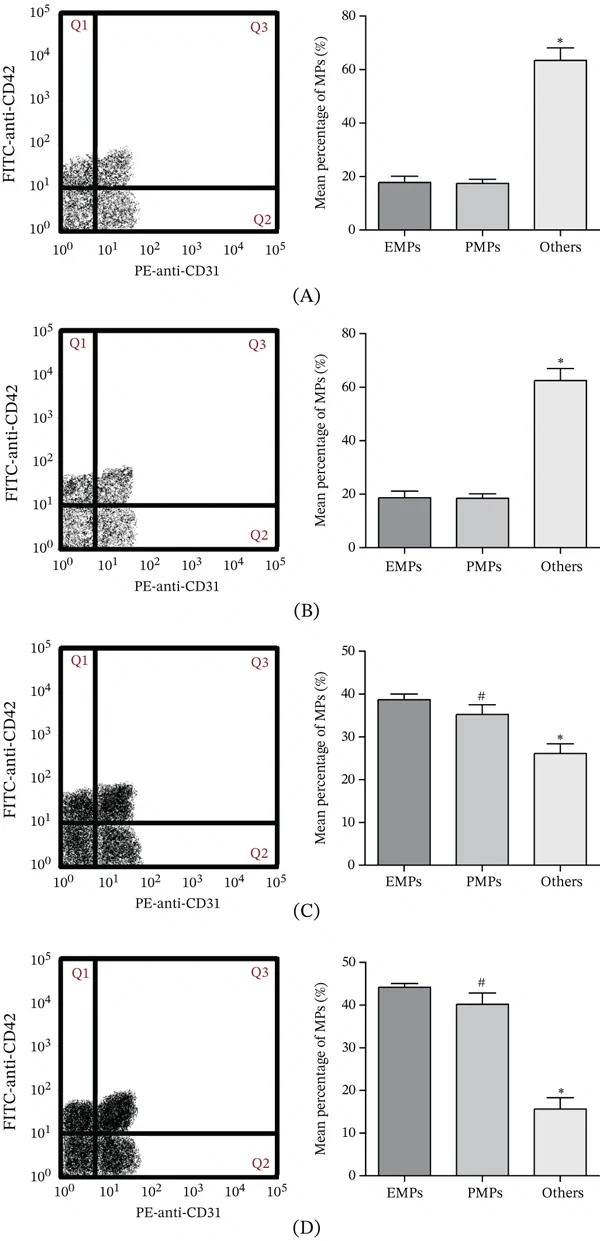
Origins of MPs. (A) Origins of MPs from healthy subjects: Few EMPs (Q2, 17.78*%* ± 2.33*%*) and PMPs (Q3, 17.44*%* ± 1.51*%*) were detected. (B) Origins of MPs from control: Few EMPs (Q2, 18.67*%* ± 2.45*%*) and PMPs (Q3, 18.44*%* ± 1.67*%*) were detected. (C) Origins of MPs from STEMI: EMPs (Q2; 38.67*%* ± 1.32*%*) and PMPs (Q3; 35.22*%* ± 2.28*%*) significantly increased. (D) Origins of MPs from STEMI + NRF: EMPs (Q2; 44.18*%* ± 0.87*%*) and PMPs (Q3; 40.18*%* ± 2.64*%*) further increase.  ^∗^ *p* < 0.05 versus EMPs and PMPs, # *p* < 0.05 versus EMPs.

Compared with healthy and control (Figure 8A,B, few EMPs and PMPs were detected), EMPs (Figure 8C, Q2; Figure 8D, Q2) and PMPs (Figure 8C, Q3; Figure 8D, Q3) accounted for the majority of the MPs in STEMI and STEMI + NRF. These results demonstrated that both EMPs and PMPs may play the main functional role of MPs. As there was no method to isolate EMPs/PMPs from the blood at present, we used MPs in our experiments.

## 4. Discussion

Our study revealed that MPs from STEMI patients (especially those with NRF during PCI) inhibit eNOS activation and vasodilation and activate oxidative stress and inflammatory responses in experimental models. Our results suggests that MPs may play a detrimental role in STEMI patients who develop NRF during PCI.

Current research indicates that MPs participate in the regulation of inflammatory responses, coagulation function, vascular dysfunction, and vascular regeneration and are implicated in the onset and progression of multiple vascular‐associated diseases [17, 18].  ^∗^Circulation Research ^∗^has published multiple thematic reviews focusing on MPs, which cover intracellular signal transduction, angiogenesis, vascular function, and atherosclerosis [17–20]. The pathophysiological processes underlying STEMI and NRF encompass inflammatory responses, platelet adhesion and aggregation, and endothelial dysfunction. These pathophysiological alterations can trigger coronary artery spasm and thrombosis and may further induce an elevation in the concentration of circulating MPs [1–4]. The present study observed that the concentration of MPs in STEMI patients, particularly those experiencing NRF during PCI, was significantly higher than that in healthy controls and patients with angina pectoris and negative coronary angiography results. Based on this finding, we hypothesize that MPs may not merely serve as diagnostic markers for heart disease but are also likely to related to the severe complication of NRF occurring during PCI.

The mouse aorta, which has long been used as a substitute for human vascular tissue, is an established model that bridges the gap between in vitro and in vivo studies (including those on atherosclerosis, hypertension, metabolic pathways, aortic aneurysms, vasoconstriction, and vasorelaxation) [21].

In the present study, under equivalent doses of MPs across all experimental groups, we observed that MPs isolated from patients with STEMI (particularly from patients who developed NRF following PCI) exerted a significant inhibitory effect on endothelium‐dependent relaxation. Furthermore, preincubation with L‐NAME largely reversed the inhibitory effect of these MPs, which indicates that MPs impair endothelium‐dependent relaxation primarily through pathways mediated by eNOS in experimental models. In subsequent research, we will verify the above regulatory effect through genetic (experiments using eNOS knockout mice and nucleic acid, intervention experiments including gene silencing and overexpression). Meanwhile, we also plan to conduct follow‐up experiments with other pathway inhibitors to exclude the contribution of non‐eNOS pathways and further validate the specific mechanism through which MPs exert their biological effects.

Deletion of the caveolin‐1 gene is known to induce functional abnormalities in the cardiovascular system. Caveolin‐1 can directly bind to eNOS and exert negative regulatory effects, maintaining eNOS in an inactive quiescent state. This negative regulatory effect of caveolin‐1 on eNOS subsequently leads to a reduction in NO production [22]. In the current study, we observed that MPs isolated from patients with STEMI (particularly patients complicated with NRF during PCI), significantly upregulated the expression of caveolin‐1. Based on this finding, we hypothesize that MPs‐induced upregulation of caveolin‐1 expression may promote eNOS inactivation, thereby ultimately inducing endothelial dysfunction in experimental models. Nevertheless, further verification of this hypothesis via experimental studies using targeted intervention tools for the caveolin‐1‐eNOS interaction (e.g., specific inhibitors) is still required.

eNOS is an important source of NO produced by endothelial cells and a crucial regulatory substance for maintaining cardiovascular balance [23–27]. The balance between NO and O_2_^•−^ plays a key role in regulating cardiovascular function; disruption of this balance can lead to the occurrence and development of multiple cardiovascular diseases.

The present experimental models study confirmed that following treatment with MPs isolated from patients with STEMI, especially MPs collected from STEMI patients complicated with NRF during PCI. The phosphorylation level of eNOS at Ser1177 site and the production of NO decreased significantly, whereas the phosphorylation level of eNOS at Thr495 site and the production of O_2_^•−^ increased significantly. Combined with the findings of this study and existing research conclusions from other scholars [7, 8, 28, 29], we propose that the mechanism of MPs‐induced endothelial dysfunction is that MPs reduce eNOS‐dependent NO release, which ultimately leads to impaired endothelial function. Future studies will use eNOS knockout mice to verify the above conclusions.

ICAM‐1 is an inflammatory mediator closely correlated with coronary heart disease, which mediates the adhesion of inflammatory cells to the vascular endothelium and facilitates the initiation and progression of inflammatory responses [30]. In line with this biological function, we detected the expression level of ICAM‐1 to investigate whether MPs affect endothelial function through inflammatory pathways. The results of the present experimental models study demonstrated that MPs isolated from STEMI patients, particularly those from patients experiencing NRF during PCI, significantly upregulated ICAM‐1 expression. This finding indicates that MPs may be associated with the occurrence of NRF during PCI by promoting inflammatory factor‐mediated adhesion in the experimental model. Although the present study did not directly examine the effect of MPs on inflammatory cell adhesion in vivo and in vitro, this underlying mechanism can be reasonably inferred based on established conclusions in existing literature [30].

Coronary collateral growth, which is primarily characterized by angiogenesis, plays a critical role in the pathophysiological process of STEMI. Although collateral angiogenesis cannot be acutely upregulated to provide myocardial protection during the acute phase of STEMI, it is a key regulator of postinfarction myocardial healing and ventricular remodeling [31, 32] and may substantially influence the long‐term prognosis of affected patients. In the present study, we observed that MPs isolated from STEMI patients, particularly those isolated from STEMI patients complicated with NRF after PCI, significantly inhibited the proliferation and tube formation of HCAECs. These findings suggest that MPs are not only correlated with the occurrence of NRF during PCI in STEMI patients in experimental models but may also exert an important impact on the long‐term prognosis of STEMI patients.

L‐NAME, a NOS inhibitor, is able to induce NO deficiency, inflammatory responses, oxidative stress, and endothelial dysfunction [33]. In the present study, L‐NAME was observed to partially block the promoting effects of MPs on the proliferation and tube formation of HCAECs, which indicates that additional mediators may also participate in the regulatory effects of MPs on endothelial cell proliferation and tube formation.

### 4.1. Limitations

Owing to the relatively small sample size in the present study, particularly with respect to subgroup analysis, further large‐scale multicenter clinical and basic research is warranted to verify the reliability of the results and conclusions obtained in this study. Furthermore, the present study is not a randomized controlled clinical trial, and the sample size was not determined via a priori statistical power calculation, as this investigation only constitutes basic research that extracts MPs from patients′ peripheral blood and explores the effect of NRF on the function of MPs. Excluding patients with specific comorbidities may restrict the external generalizability of the study findings. Additionally, given that the present study employed mouse tissues, interspecies biological heterogeneity constitutes another key limitation of this investigation. In addition, inadequate control for confounding factors (such as door‐to‐balloon time, duration of NRF, thrombus burden, infarct location, lesion complexity, and peak troponin) is another weakness of this study. Meanwhile, pooling of isolated MPs derived from the same study group may reduce the sensitivity of research findings, as this procedure eliminates interpatient variability, decreases statistical independence among observations, and precludes the exploration of correlations between MP levels and observed phenotypes. Therefore, future research should be conducted based on individual samples. In the current investigation of ICAM‐1 expression, cell proliferation and tube formation, the absence of functional blocking experiments (including neutralizing antibody intervention targeting candidate surface proteins and RNase or protease pretreatment of MPs) reduces the robustness of the established causal association between MPs and the observed biological phenotypes. Furthermore, complementary mechanistic validation experiments, including experiments based on eNOS knockout mouse models and siRNA knockdown in HCAECs (including caveolin‐1 inhibition, antioxidant intervention, and ICAM‐1 functional blocking) are required to further confirm the underlying molecular mechanism. Finally, given that both the STEMI group and the STEMI + NRF group presented elevated lipoprotein levels and received pharmacologic interventions including statins, antiplatelet agents, and beta‐blockers, whether lipoprotein levels and pharmacologic treatments confounded the study results remains undetermined. Future studies will be conducted to systematically explore the effects of lipoproteins and pharmacologic interventions on MPs.

In summary, the present study demonstrates that MPs isolated from patients with STEMI (particularly from those complicated by NRF during PCI) negatively modulate vascular function via multiple mechanisms in experimental models. They inhibit eNOS activation, endothelium‐dependent vasodilation, HCAECs proliferation, and capillary‐like tube formation while simultaneously inducing oxidative stress and promoting the adhesion of inflammatory factors. Our findings further reveal that MPs exert detrimental effects on cardiovascular function and may adversely affect the long‐term prognosis of STEMI patients post‐PCI in experimental models. As MPs consist of proteins and nucleic acids and mediate intercellular information transmission, they hold great potential to serve as novel clinical biomarkers or therapeutic targets for cardiovascular diseases. Currently, owing to the heterogeneous nature of MP‐derived protein and nucleic acid components, no specific neutralizing agents targeting the entire MP population are available. Nevertheless, future studies can develop targeted intervention strategies against specific functional proteins or nucleic acids carried by MPs.

## 5. Conclusion

Based on the findings of this study (associational in the clinical context and mechanistic only in experimental models), the application of MPs is anticipated to deliver the following clinical values in clinical practice: For patients who present with elevated MPs levels following PCI, clinicians are recommended to adjust therapeutic regimens to inhibit oxidative stress and inflammatory responses and improve vasodilatory function, so as to ameliorate the long‐term prognosis of patients.

Furthermore, in future research, we will test the MPs content of STEMI patients with or without NRF before PCI to investigate the relationship between MPs and NRF. This work may yield greater clinical significance and support broader translational applications.

## Author Contributions

F‐J.C.: conceptualization, methodology, supervision, writing—reviewing and editing. W.Z., W.L., and Y‐J.Y.: data curation, investigation, writing—original draft preparation, software. Z.L., X.J., and G.C.: writing—original draft, formal analysis, resources. W.Z. and W.L.: data curation, supervision, writing—original draft. G.C.: funding acquisition.

## Funding

This work was supported by the Science and Technology Talent Support Program Funding Project of Shaanxi Provincial People′s Hospital, No. 2022LJ‐04.

## Ethics Statement

Informed consent was obtained from all participants and/or their legal guardians (mostly used when patients are illiterate). The research was approved by Shaanxi Provincial People′s Hospital (The Third Affiliated Hospital of Xi′an Jiaotong University) Ethics Review Board. Experiments involving animals were approved by The People′s Hospital of Shaanxi Province (The Third Affiliated Hospital of Xi′an Jiaotong University) animal ethics committee.

## Consent

Informed consent was obtained for the publication of patient data.

## Conflicts of Interest

The authors declare no conflicts of interest.

## Data Availability

Data available on request from the authors.
